# Research on mobile learning platform interface design based on college students’ visual attention characteristics

**DOI:** 10.1371/journal.pone.0283778

**Published:** 2023-07-07

**Authors:** Tiejun Zhu, Yujin Yang

**Affiliations:** 1 Anhui Normal University, Wuhu, China; 2 Anhui Polytechnic University, Wuhu, China; Jeonbuk National University, REPUBLIC OF KOREA

## Abstract

To explore the visual experience characteristics and influencing factors of college students’ visual attention intervention in the interface of mobile learning platform by using eye-tracking technology, and to summarize and summarize the visual experience pattern of platform interface design and its design inspiration. Methods: Using the head-mounted eye-tracking technology, 28 images from 6 groups of typical elements in the interface layout of CGTN learning platform were selected as the test samples, and the eye-movement indexes of the subjects browsing the interface were recorded. Results: There were significant differences in the attention time, number of times of attention, visual attention rate and visual recall rate of different areas and topics of the interface (P < 0. 001). Conclusion: In the platform interface design, the analysis of the factors influencing visual attention can be found that people’s attention and visual experience is mainly influenced by color, text, and typography, and secondary areas and layout also play an important role in visual communication. The color and text areas in the interface design, as well as the innovative design of typography can effectively enhance the visual attention of college students and better communicate the information of the platform.

## Introduction

UI interface design has an important role in identifying and guiding functions in the Internet era. With the development of the Internet, interface design has not only been limited to simple recognition and formal aesthetics, but has gradually improved to both usability and ease of use. From the perspective of interface cognition, interface includes physical features and comprehensive features. Physical features refer to the external expression of the elements in the interface, such as icons, including the background, line, size, shape and color of the icons [1]. Integrated features include the figurativeness, complexity, familiarity and semantic distance of the interface. In the visual search of the device interface, the features of the interface elements can influence the visual attractiveness and the search efficiency of the user, thus affecting the user experience of the interface operation. The accuracy and scientific nature of eye-tracking technology in visual attention measurement makes it an important tool for revealing visual attention, measuring individual visual processing and becoming an important tool for interface research. Using this technology, it is possible to derive eye-movement indicators such as user’s gaze time and number of gaze to each area in a certain time period, thus becoming a key area to solve the problem of visual attention of college students in the process of interface propagation. Therefore, the interface design must be considered from both physical features and comprehensive features. Visual attention itself is a complex cognitive process, and the attention task also requires different attentional processing to complete, and the factors affecting visual attention in this process can be different. This paper explores the influence of UI interface elements on visual attention based on theories related to visual attention, taking the visual cognition of college students in the interface design as the research object. Through eye-movement experiments, we examine the influence of variables on relevant indicators at different stages of gaze, and combine the cognitive mechanism in visual search to reveal the influence of UI interface design elements on college students’ visual attention, so as to improve smart user experience and ease of use. It will be of guidance to the development and design of the interface platform of mobile learning platform.

## Related work

### Analysis of UI design elements

#### User-centric

*Using the user language*. Interface design should adhere to the user-centered, using the language of the user, rather than the language of the designer, in learning to combine specific software requirements analysis, fully consider the user’s desired combination of information and interface presentation effect.

*Application-compliant scenarios*. When designing the interface, we should do full research and fully consider the basic characteristics of the carrier. The overall design of the interface should consider the combination with the carrier scene, actively adapt to the application scene and select things associated with professional applications or representative icons, colors, and layouts as the design elements of the interface.

*Timely information alerts*. In the process of user operation of the software, to provide timely feedback to the user-friendly information prompts, but also to give timely warnings, tips, and other information. For example, the software in the processing of a large amount of data tasks, often can not immediately return the results, when waiting for the system feedback time needs to be set to the user "is processing, please wait" information prompts or progress status bar graphic prompts, can not feedback to the user a non-responsive interface [2]. Another example is the software in the processing of information and data, sometimes the operation is irreversible, at this time must be indicated to the user "irreversible operation" of the relevant tips to facilitate the user to do a good job of data backup.

#### Artistic aesthetics

*Color Harmony*. Software interface, color is often more impactful than graphics, text, etc. To the user, so the main color of the software should also match the scene served, such as environmental protection class recommended using green tone design to reflect the meaning of environmental protection; if the police, you can use police blue and white as the main color. If the color chosen for the interface is extremely incompatible with the application scenario, it will look out of place and difficult to be recognized by users [3]. In addition, it is also crucial to select the right number of colors and match them harmoniously in the interface design. In terms of color selection, the number of colors should be too many, generally no more than three color families, because too much color accumulation will make the interface look dazzling. In terms of color matching, there will be multiple display elements such as text, tables, and icons in the system at the same time, and the harmony between colors should be fully considered in the interface design to avoid clutter.

*Aesthetically pleasing design*. The interface should uphold the principle of beautiful design, the interface of the software is beautiful or not, directly affects the mood of the user, and within u, r n affects the intuitive impression of the software. For example, the login page of the software is the first interface that users see when they login to the system, and it needs to be beautifully designed to attract the users’ eyes.

*Reasonable layout*. Each element of the software interface should be reasonably laid out, taking the common operation window as an example, the window size and placement of the search bar, toolbar, the status bar, and layer list bar should be careful [4]. The software is for the user, so the window should not be set too small to affect the user’s reading and use, nor should the window be set too large to affect the user’s browsing and operation in the area. The elements of the interface are laid out in a reasonable manner, which naturally promotes the beauty of the interface and is a reflection of the principle of artistry.

#### Consistency principle

*Consistent style pattern*. Consistent style is reflected not only in the uniformity of the style of each window within the system but also in the uniformity of the style of the design elements. Such as the common toolbar in the software, there are generally several common tools such as search, layer, mark, clear, print, etc., In the design of the tool button, to use a unified style, that is, the selected representative function of the icon style to be consistent, or unified flat style, or unified three-dimensional style, or unified anthropomorphic style, etc., Not in the same software mixed with different styles [5]. In the same style, you can make the interface look more beautiful and generous, to a large extent, to enhance the artistic level of the interface.

*Consistent interface structure*. The software often provides multiple functions that are similar in operation but implemented by different spatial analysis algorithms, i.e., each function is implemented differently, but as much as possible to summarize the common denominator, and strive to make all functions have approximately the same interface structure, the consistency of the interface structure is reflected in each step of the operation, but also reflected in the operation window interface. The consistency of the interface structure so that users have predictability in the operation of the software can help users get started faster, that is, once the user has mastered the use of one of the functions of the current software, you can master the operation of other functions using the same interface structure [6].

#### Efficient principle

*Simple interface presentation*. The meaning of simplicity is reflected in the user’s preference for simple and regular graphics, on the other hand, the characteristics of simplicity and rules can make the user’s operation simple and procedural. Thus, the concept of "simplicity" should be used throughout the design of the software interface, and the interface should be clear and concise when it is finally presented to the user, to reduce unnecessary interference to the user and avoid misuse during user interaction.

*Easy user interaction*. Software interface design should be designed to facilitate the operation of the user as the goal, convenient interface design will improve the efficiency of the use of the software [7]. Such as the availability of a menu to complete multiple processing links, should not be designed separately for multiple steps, such as in the same window must be completed by more than one menu operation, and should not be associated with the location of the menu isolated far away.

*Accurate transmission of information*. The information displayed on the interface, such as text or graphics, should match exactly with the actual functions pointed to, to deliver accuracy and avoid ambiguity. As a user, the user does not understand or needs to understand the logic of code-level implementation, so the designer should always consider the presentation of the interface from the user’s operation perspective. Therefore, the designer should always consider the presentation of the interface from the perspective of the user operation. There is both the reaction of the instinctive level of user experience and the embodiment of the behavioral level of user experience. Second, the degree of visual attention.

## Methods

### Visual attention span

Attention is an important component of cognitive psychology, which refers to the concentration of mental activity that is accompanied by continuous eye movements. Usually, attention tasks require both bottom-up and top-down processing [8]. Top-down processing refers to the deceiver’s learned experiences, expectations, and motivations that guide the deceiver’s selection, integration, and construction of information and representations during perception, such as when Percival performs a visual search task [9]. In contrast, the stimulus-driven alternation selection is bottom-up processing, also known as transient exogenous attention. In visual attention, sometimes a focused mental activity is actively taken, which implies a desire to attend to a stimulus (top-down processing). For example, when looking for a target icon in a set of interfaces, one will focus on features related to the target (color, shape, size, etc.). Sometimes particular features of a stimulus are attended to because the interesting stimulus captures attention (bottom-up processing). For example, in a group of interfaces, the one with a special shape tends to be noticed more. In visual search, visually appealing targets are often more frequently looked at. It has been found through research that people’s attention can be drawn to image elements and that different types of images lead to different forms of gaze duration, intelligence, and visual learning.

### Visual complexity

Visual complexity is often defined as the level of detail or complexity contained in a picture [10]. In interface research, complexity is defined as the number of basic shapes that make up an interface, including the number of shapes and lines, etc. Feature integration theory proposes that simple icons are easier to be understood than complex ones [11]. For example, Den Dong ET AL. Found through experiments that flat icons is simpler and more efficient than anthropomorphic icons, which are more attractive to users’ attention and have higher cognitive efficiency. However, in visual search tasks, when familiar icons are considered simpler, the search efficiency of simple icons does not show a significant advantage [12]. Third, eye movement experiment studying the visual attention and comfort of college students through design elements, we analyzed the whole process of "eye skip(sweep)—gaze—stare" using an eye-tracking device to obtain the user’s visual attention and emotional comfort needs, as well as the evaluation criteria for design. The study will also use the UI web pages to evaluate the design of the web pages. The study will also evaluate the implicit measurement of interface design elements of UI web pages to capture the "subconscious behavior" of users. Due to the different purposes and subjects, implicit measurement evaluation includes various methods such as eye movement tests, EEG tests, and functional MRI. The study selects the eye-movement test, which is a relatively simple experimental equipment and intuitive data performance, to capture the unconscious behaviors of users in the process of observing interface design elements, and summarize and generalize the visual experience pattern and the comfort level. First, the official website interface of the English language, which is more authoritative and representative among web pages, was selected as the test sample and grouped, and the subjects were subjected to eye-tracking experiments using on-screen eye-movement test equipment; then, the visual experience characteristics of different elements of web pages such as color, layout, font, and font color were evaluated in groups, and the most afterward, additional interviews were conducted for the subjects to understand their subjective feelings and visual comfort, and to synthesize the visual attention and comfort experienced in web design elements.

## Experimental

### Experimental equipment

The **Tobii TX300** eye-tracking device was used to capture human eye movement behavior and attention focus through eye movement patterns, and to use them as objective indicators. The eye-tracking device was connected to a computer monitor, and its program, color temperature, brightness, and contrast were adjusted consistently to ensure that the subjects were sitting in front of the monitor, and the observation distance was controlled at 60–80 cm. The software analysis was used to adjust the point-of-view path, line graph, zone of interest sequence, gaze graph, heat zone graph, and other indicators of the subjects’ eye movements. Through the above indicators, we can understand where and for how long the subjects would observe first when observing the web design elements, and with the help of eye movement indicators such as eye gaze time and number of gaze in the observation area, we can infer the point of interest of people to the interface and its reasons.

### Subjects

The target subjects of the experiment were selected to be **30** related to the design profession. To prevent practice effects, none of the experimental subjects were exposed to the test samples in advance, and none had visual or color impairment.

### Experimental materials

The web pages of CGTN, a typical and authoritative website on the market, were selected as a sample for observation through research and classified into different modules according to different design elements: title, layout, font, font size, the font color, the interface color, and links.

The subjects’ sitting posture and the eye condition were first ensured, and then a combined eye-movement test was conducted on the sample images for comparison. The subjects were asked to browse the images of the given web design elements according to their daily habits through textual prompts in the interface and pressed the space bar to continue until the end. Combining the eye-movement experiment data and the interview data, we obtained their eye-movement hot spot diagram, path diagram, gaze diagram, and experiment data to understand the subjects’ visual concerns, to summarize the visual experience characteristics and visual comfort of the interface design elements, and propose practical discussions on the design application. During the experiment, the testers tracked the subject’s eye-movement information throughout and made adjustments to the abnormal situation. The number of gaze points, average gaze time, line of sight gaze rate, first gaze time, and line of sight return rate of each experimental sample were collected from the experimental data, and the experimental data were compared, analyzed, and interpreted to explore the influence of interface design seriousness on college students’ visual experience.

## Experimental results and analysis

**Fig 1:**
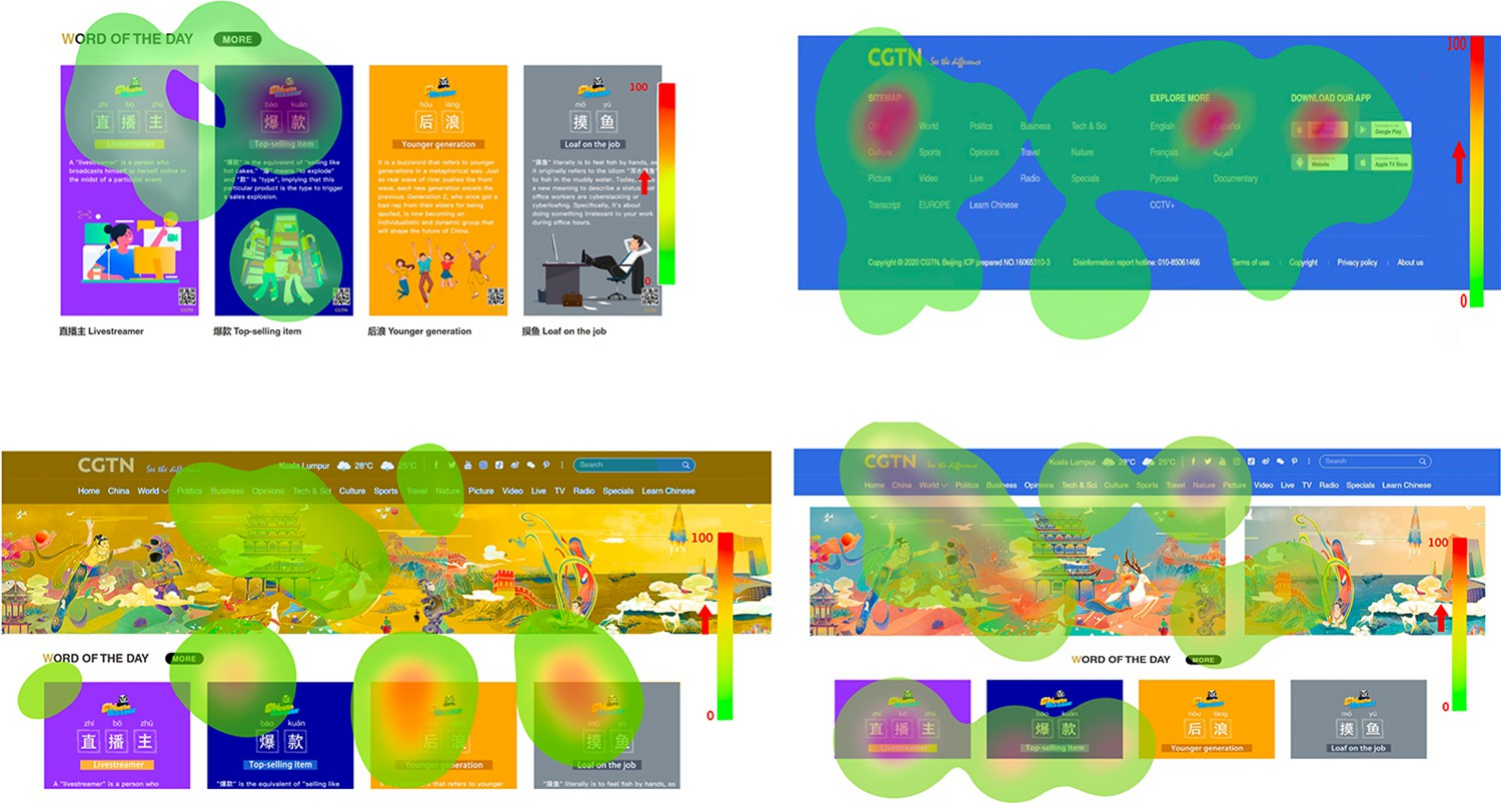
Thermal zone map of eye movement experiment.

For example (Fig 1), the larger the circle in the path diagram, the longer, and more times the subject looked at the point. The warmer the color, the longer the time spent on the area, and the more attractive the area is to the subjects, thus finding out the highlight areas of the interface [13]. Through the analysis of the gaze points, the average gaze duration, and the return rate of sight, we found that the font was mainly in sample No.1 Times New Roman; the font color was mainly in sample No.5 Blue; the headline was mainly in sample No.1 Large; the layout was mainly in sample No. 1 Banneker the layout; links are mainly focused on sample link 1 without underline; page color is mainly is concentrated in the green page of sample No 4. Table 1 describes each reference index of the eye movement experiment and explains them as well as the criteria for evaluating the experimental data; Table 2 shows the results of the experimental data analysis between the experimental material and the eye movement index.

**Table 1:** Reference indicators and evaluation criteria.

Reference Indicators	Evaluation Criteria
**Number of focus points**	The higher the number of attention points, the more information the sample can provide to the subjects
**Average gaze duration**	The higher the gaze duration, the richer the intention shaped by the sample in the subject’s mind
**Sighting rate**	The ratio of the number of people observing this sample to the total number of people in the subgroup experiment
**Knightliness Rewind Rate**	The higher the look-back rate, the greater the subject’s interest in that sample

**Table 2:** Eye movement experiment data.

Group	Sample No.	Number of attention points (total)	Average gaze duration/ms	Sight gaze rate/%	Sighting back rate %
Fonts	1	22.56	5004.78	100	0.78
	2	12.44	2283.22	100	1.21
	3	13	3168.89	100	0.65
	4	20	4319	100	0.79
	5	16.22	3818.78	100	0.99
	6	9.44	2243.889	100	0.62
Font color	1	11.67	2308.67	100	1.20
	2	8.22	2146.44	100	0.74
	3	8.22	2033.67	100	0.96
	4	11.33	2778.67	100	0.57
	5	13	3292.11	100	0.71
Title word	1	2.44	488.3704	100	0.73
	2	1.93	346.7037	100	0.59
	3	1.3	231.6296	100	0.51
Layout	1	17.78	4183.875	100	0.46
	2	8.44	2065.778	100	0.79
	3	8.89	2161.667	100	0.59
	4	7.22	1863.889	100	0.50
	5	9.78	2310.667	100	0.56
Link	1	17.78	4183.875	100	0.79
	2	8.44	2065.778	100	0.79
	3	8.89	2161.667	100	0.56
	4	7.22	1863.889	100	0.50
	5	9.78	2310.667	100	0.53
Page color	1	9.56	2121.556	100	1.25
	2	8.78	1975.889	100	0.53
	3	9.67	2094	100	0.63
	4	14.3	3189.889	100	0.76

In Table 3, correlation analysis was used to investigate the correlation between the experimental material and the subjects’ mean gaze time, the rate of sight recall, and the mean gaze area, and the Pearson correlation coefficient was used to indicate the strength of the correlation. The correlation coefficient between the experimental material and the mean gaze time was 0.942 and showed a 0.01 level of significance, indicating that the correlation between the two was significant.

**Table 3:** Pearson correlation analysis.

	Average gaze time/ms	Sighting back rate %	Average gaze area
**Pearson Correlation**	.942	.876	.640
**Significance (two-tailed)**	.000	.000	.000

** Significant correlation at the 0.01 level (two-tailed).

**Statistical reporting.** *Total number of gazes*. On the total number of gazes, repeated measures ANOVA results showed a significant main effect of difference between interest areas, F(4,116) = 4.423(Table 4), p<0.01, and post hoc tests showed significantly more total gazes in the font size interest area than in the link interest area (p<0.05), with no significant differences between the remaining interest areas (all p>0.05).

**Table 4:** Analysis of variance table for total number of gaze.

Source of effect	Methods	Square and	Degree of freedom	Mean Square	F	Significance
**AOI**	Assuming sphericity	6727.492	4	1681.873	4.423	0.002
	Greenhouse Geisler	6727.492	2.792	2409.902	4.423	0.007
	Sin-Fedt	6727.492	3.119	2156.83	4.423	0.005
	Lower limit	6727.492	1	6727.492	4.423	0.044
**Error (AOI)**	Hypothetical sphericity	44106.36	116	380.227		
	Greenhouse-Geisler	44106.36	80.957	544.816		
	Sin-Fedt	44106.36	90.456	487.603		
	Lower limit	44106.36	29	1520.909		

*Average gaze duration*. On the mean gaze duration, the repeated measures ANOVA showed no significant difference in the main effect of interest area, F(4,116) = 0.964(Table 5), p>0.05, and therefore no post hoc comparison between interest areas was necessary.

**Table 5:** Mean gaze duration ANOVA table.

Source of effect	Methods	Square and	Degree of freedom	Mean Square	F	Significance
**AOI**	Assuming sphericity	2.79E-05	4	6.96E-06	0.964	0.43
	Greenhouse Geisler	2.79E-05	3.158	8.82E-06	0.964	0.417
	Sin-Fedt	2.79E-05	3.589	7.76E-06	0.964	0.424
	Lower limit	2.79E-05	1	2.79E-05	0.964	0.334
**Error (AOI)**	Hypothetical sphericity	0.001	116	7.23E-06		
	Greenhouse-Geisler	0.001	91.58	9.15E-06		
	Sin-Fedt	0.001	104.071	8.06E-06		
	Lower limit	0.001	29	2.89E-05		

*Average pupil diameter*. On the mean pupil diameter, repeated measures ANOVA results showed a significant main effect of difference between interest areas, F(4,116) = 10.779(Table 6), p<0.001, post hoc test results showed that the mean pupil diameter of the title interest area was significantly larger than that of the font interest area (p<0.05), the mean pupil diameter of the layout interest area was significantly larger than that of the font interest area (p<0.001), the link interest area (p<0.001), the mean pupil diameter of the font size interest area was significantly larger than that of the font interest area (p<0.01), the mean pupil diameter of the link interest area was significantly larger than that of the font size interest area (p<0.01), and there was no significant difference between the remaining interest areas (all p>0.05).

**Table 6:** Mean pupil diameter ANOVA table.

Source of effect	Methods	Square and	Degree of freedom	Mean Square	F	Significance
**AOI**	Assuming sphericity	0.216	4	0.054	10.779	0.000
	Greenhouse Geisler	0.216	3.086	0.07	10.779	0.000
	Sin-Fedt	0.216	3.495	0.062	10.779	0.000
	Lower limit	0.216	1	0.216	10.779	0.003
**Error (AOI)**	Hypothetical sphericity	0.582	116	0.005		
	Greenhouse-Geisler	0.582	89.486	0.006		
	Sin-Fedt	0.582	101.356	0.006		
	Lower limit	0.582	29	0.02		

*Average number of eye jumps*. On the mean number of eye jumps index, repeated measures ANOVA results showed a significant main effect of difference between interest areas, F(4,116) = 25.626(Table 7), p<0.001, post hoc test results showed that the mean number of eye jumps was significantly more in the title interest area than in the layout and font interest areas (all p<0.001), the mean number of eye jumps was significantly more in the link interest area than in the layout and font interest areas (All p<0.001), the average number of eye jumps in the font size interest area was significantly more than that in the layout and font interest areas (all p<0.001), and there was no significant difference between the layout and font interest areas (p>0.05).

**Table 7:** Analysis of variance table for the mean number of eye jumps.

Source of effect	Methods	Square and	Degree of freedom	Mean Square	F	Significance
**AOI**	Assuming sphericity	66.214	4	16.553	25.626	0.000
	Greenhouse Geisler	66.214	3.358	19.721	25.626	0.000
	Sin-Fedt	66.214	3.85	17.198	25.626	0.000
	Lower limit	66.214	1	66.214	25.626	0.000
**Error (AOI)**	Hypothetical sphericity	74.931	116	0.646		
	Greenhouse-Geisler	74.931	97.368	0.77		
	Sin-Fedt	74.931	111.652	0.671		
	Lower limit	74.931	29	2.584		

### Analysis of experimental results from font samples

In the experiment, the fonts were divided into font and color groups, and the front group consisted of six samples, namely, Times New Roman, bold, Chinese amber, Chinese lineal, Song, and italic, which were mainly distributed in the navigation bar and function bar on the interface, playing the role of page navigation and explanation. According to the experimental results, the subjects paid the most attention to the Times New Roman font of sample No.1, and according to their eye-movement data, the number of gaze points and the average gaze duration of sample No.1 were larger than the rest of the samples, and from the path diagram, the average speed of the subjects’ gaze points into No.1 was the fastest. times new Roman is a transitional serif font and is often chosen as one of the standard fonts because of its classic appearance of medium and four-sided. In the font color sample experiment, the font was used as the basic Times New Roman font, and the samples were color-changed based on the font, mainly divided into white, black, red, yellow, and blue. By analyzing the eye-movement data, the subjects we’re interested in the white font for sample No.1, the yellow font for sample No. 4, and the sample has a higher attention span in blue font, and the path diagram shows that the subjects’ eyes enter sample #5 when, therefore, the subjects’ visual attention to the blue font number 5was the highest when combined with the experimental data. The color of the blue font is the sky blue of CMYK standard color, also known as blue, its wavelength is between 485-470nm, between blue and cyan, similar to the color of a clear sky [14]. Sky blue represents tranquility, freshness, and freedom, and some studies show that this blue color is a soothing color that makes people feel comfortable visually and relaxed, and free mentally. In general, people are visually more comfortable with standard, easy-to-understand serif fonts, and colors in the online platform interface, and the visual attention of font colors with saturation and brightness in the middle is higher.

### Analysis of experimental results from the title word sample

In the headline sample experiment, the font of the headline was chosen as the standard font black, the color of the font was chosen as black, and the size of the headline was taken as the variable to explore which size of the headline was the most attractive to the subjects’ visual attention. From the path diagram, the highest value of the headline dwelled in the No. 1 sample group; from the hot spot diagram, the subjects’ hot spots radiated to the sides with the central axis as the visual center, and then radiated from the central axis or the central point to the sides and all around, and the hot spots were basically in the area of large text headlines; No. 2 sample’s visual center point first fell in the area of large text headlines. The visual Center Point of sample No. 2 first fell on the middle large text, and the subjects would first notice its enlarged title name, and then expand their vision outward from this center, and observe other surrounding text and patterns. The large caption set was set with a font size of 70 points, with a spacing of 15, and a bold setting. A large title word plays an important role in emphasizing and highlighting the whole platform interface and makes the interface information clear and easy to read, with a clear hierarchy, so that users can capture the information faster [15]. On the contrary, an unreasonable title size will make the interface confusing and affect the reading experience. On the whole, a large title word in the online learning platform interface can attract users’ visual attention.

### Analysis of experimental results from interface layout samples

In the interface layout experiment, mainly divided into the interface home page layout form samples and interface link layout form, the layout is mainly divided into Banner type layout, POP type layout, column layout, and corner type layout In the layout experiments, the highest attention point (19.4) fell on sample No. 1, and the highest sight return rate was found in sample No. 2; combined with the experimental results such as the sight heat zone map, the visual attention of Banner-style layout of sample No. 1 was the highest, and when subjects viewed sample No. 1, their sight was mainly focused on the upper half of the title interface and the lower half of the sub-column interface, and the overall was in the middle of the page. When the subjects viewed sample No. 1, their eyes were mainly focused on the upper part of the title screen and the lower part of the sub-column screen, which were in the middle of the page. The advantage of this layout is that it makes the structure of the interface look clearer and more prioritized, makes the page neat and stable, eye-catching and generous, more visually focused, and makes the overall interface have a certain impact while highlighting the main subject; and this layout is not limited by the size of the platform. interfaces restrictions. In the link layout, the color, font, and typography of the links are used as quantitative, mainly divided into four styles: no underline dashed underline, straight line with arrow underline, and straight-line underline, to explore which link form is more preferred by users [16]. In the link experiment, the subject’s attention was mainly on sample No. 1 without underlining, and the sample #1 and Sample #2 have a higher rate of line-of-sight return; Sample #1 is relatively simple and generous in terms of color, layout, typography, and text, giving the most comfortable visual effect. This type of link without underlining is more concise, avoids useless information interference, greatly facilitates users’ search and reading, and improves users’ access time and access efficiency. On balance, the Banner layout and the link layout without underlines are the most visually appealing to users.

### Analysis of experimental results from layout color samples

In the layout color sample experiment, which was mainly divided into four colors: black and white color, red, yellow, and green, in the page color, the subjects’ visual path first fell on the area with relatively sharp color. In the 4 groups of samples in this book, subjects had a high rate of return viewing in sample #1, and the highest gaze point and average gaze duration in sample #4green. The neutral green color chosen for the page belongs to the color of nature. This color gives people the feeling of vitality and revival, which makes users effectively reduce visual fatigue when reading, feel relaxed and at ease, increase the sense of intimacy and pleasure, and are full of hope and youthful vitality [17]. Overall, the user’s visual attention is mainly on the page color green.

By comparing the path diagrams and gaze diagrams of the eye-tracking experiments designed by the online learning platform, we found that almost every subject’s visual center point fell products with higher color brightness or more obvious contrast first. The gaze diagram shows that the experimental samples all exhibit sub-hot spots scattered bright spots, suggesting that the subject fragmented the key points of the experimental material to obtain information. It can be seen that the text headline area is the focus area of subjects’ experiments, while other subheadings, layouts, patterns, etc., become fragmented areas that attract attention. From the overall experimental group data, the subjects’ sight points fell first on the areas with unique colors and larger and denser textures, which can be inferred that in terms of text, people first focus on the font of the text, followed by the color and size of the text; in terms of color, green interfaces are more interesting than red and yellow; people prefer typical, simple in terms of layout and links, people prefer the typical, simple page layout, which allows quick and clear access to information. In summary, the subjects have high visual sensitivity to the elements of the mobile learning platform interface design, such as title, text, color, layout, and typography, as well as interface size and layout order. When carrying out the design, the interface can be highlighted through layout position, font size, symmetry, and other design mean to attract users’ attention; with the help of reasonable patterns and symbols to strengthen memory. Pay special attention to the hierarchical processing of interface information, consciously guiding users to see the navigation bar and home page content of the interface first, and then to understand the secondary content such as the learning section, and try not to use average text Size and typographic order. Avoid redundant and distracting information. Unnecessary patterns, symbols, or text descriptions in the interface design may interfere with the visual communication of key information and affect the user’s visual attention and visual experience.

## Discussion and analysis

### Optimizing the effect of color on visual attention

In the eye-movement experiment, subjects responded to sample number5 in the font color group with more vivid colors, page color is clear that elements with higher color saturation or contrast have a significant advantage in attracting users’ visual attention, indicating that color in interface design has a positive impact on increasing visual attention rates. In interface design, color, layout, and type are considered to be the 3 most common design elements. Among them, color is the most effective way to achieve visual information transmission and is an important language in design [18]. Based on the results of the eye-movement experiment, designers can redesign the abstract emotion of color by skillfully combining color, interface, and user’s memory and association, and transforming the lightness, purity, and chromatic of color into a psychological language representing human emotion, which helps to build a specific visual experience and interface platform image. At the same time, according to the experimental line of sight entry priority, it can be seen that samples with outstanding attention attraction usually have a higher line of sight priority. Designers can consider deep excavation and accurate positioning of the interface brand image and brand background when designing, and use this to guide color matching and application to fit users’ attention habits and attention interests, to effectively convey the platform imagery and its attributes to users. Usually, the main color of the interface should be based on the color used in daily life, with enhanced or reduced saturation and contrasting colors [19]. For the platform, it should highlight and strengthen the inherent characteristics and visual features of the platform interface. For the platform, it should mainly advocate the style image of comfort, green, health, and nature. The designer can choose the colors in line with the platform image as the base tone, with soft and friendly auxiliary colors and embellishment colors, and combine the interface layout and typography form, to highlight the unique design concept of the platform. For example, the interface design that advocates high-end image can choose the Moronic color system as an auxiliary, and the interface of the platform that tends to be young can choose the popular color of the season to embellish, and the selection of color is also one of the main ways for designers to create the visual charm and attract users’ attention.

### Optimizing the impact of text content on visual attention

From the gaze diagrams of the experiments, it can be seen that all the9 samples in the 4 groups of the font sample group and the title sample group have higher attention in the text area, which indicates that the subjects pay more attention to the text area, so it can be assumed that the text area is the focus area of the subjects experiments. The text content design in the interface design has a positive effect on prolonging users’ attention time [20]. It can be inferred from the experimental results of the text area that to improve the user’s visual attention, clear and distinct typography with appropriate fonts can be used in the text area to improve the visual logic and relevance and help users understand the interface information quickly. Proper text design and typography can convey the characteristics and culture of the brand faster and more effectively. At present, most of the interface designs on the market adopt the graphic form of combining pictures and text, and the relationship between pictures and text is roughly divided into two types: one is complementary graphics, in which the text mainly highlights the theme of the platform and attracts users’ attention; the other is integrated graphics, in which pictures and text together form the main body of the interface, and the two complement each other to enhance the overall visual experience [21]. Regardless of the combination of graphics and text, text plays a pivotal role and often requires more thought from designers. In-text innovation, the information content of the platform is redesigned by designers to optimize filtered information and allow users to access platform information more directly and effectively. The use of artistic font forms and interface backgrounds echoing each other can also present different visual experiences for users, spontaneously and effectively forming a unique visual language in their minds, thus conveying interface features and platform images. For example, text can be added in brightness and saturated colors as a way to construct the design of the interface of the mobile learning platform is a big contrast to attract the user’s eyes and highlight the content of the platform conveyed by the text information more. For the interface design of the mobile learning platform, the designer can combine the colors, appropriately reduce the fancy patterns, study more on the next part, focus on highlighting the characteristics of the platform and the content name, and use the text content to strengthen the user’s knowledge and memory of the platform.

### Optimize the impact of layout style on visual attention

In the eye-movement experiment, the visual points of the path diagrams of the layout sample group and the link sample group were mainly distributed in relatively concentrated information areas, with fewer visual dwellings in the white space and solid color areas. The results of the experiments for samples 2 and 5 in the layout group and sample 4 in the link group also showed that the overall visual elements were relatively single and the contrast of the subject, the interface style of sample No. 1 in these two groups was relatively rich. On the contrary, unlike the above three samples, the interface style of sample 1 in these two groups was relatively rich, and each element in the interface caused the subjects to stop their eyes to a certain extent, which indicates that the layout and layout design of each element in the interface design plays an active role in guiding the users’ eyes. According to the Gestalt psychology theory, people’s attention to a screen is unevenly distributed within a defined range, mainly because people’s attention is easily influenced by the induction of graphics and psychological suggestions [22]. Nowadays, there are more and more interface design styles, whether the layout is centralized, left-right symmetrical, or center-symmetrical, different typography will guide users to move their eyes in a unique form. Based on the results of user visual path analysis in eye-movement experiments, designers can pay attention to the influence of layout style on visual priority, focus on the primary and secondary arrangement in the layout design of the interface, emphasize the logical hierarchy of text and images in the center line position, to guide the visual attention order of users. Different layout styles always affect the user’s experience implicitly. It is recommended to do a simple test and survey in the ore-design stage to understand the preferences of the user group, and to design in a more targeted manner. The layout of the interface is standardized and unified. The fonts, spacing, buttons, and alignment of each area and module follow the page design specification, uniform and orderly, and the font and font size conforms to the current page standard [23]. The spacing of each functional area is too wide or too narrow, resulting in user cognitive barriers, buttons are too large or too small, and the edges are not aligned will lead to a disproportionate interface, so the layout should be reasonably arranged according to the interfaces and follow the design specifications.

### Optimize the impact of secondary areas and attention points on visual attention

In the experimental gaze map, the name and text areas show more concentrated bright spots, in addition to the positions of other design elements also show scattered bright spots. It means that after the subjects browse the main information, they also pay attention to the details such as other areas of the interface, and all these secondary areas also assume an important role in information transmission, assisting the main text and patterns to complete the communication of platform information. Especially in the era of rapid development of networks and digital media, the digital media era has created the era of the attention economy, and how to attract users’ visual attention has become the key goal of design. The way people acquire information and analyze information has also changed dramatically, forming a new visual behavior pattern -continuous partial attention, often also called attention fragmentation [24]. People subconsciously filter the information they want to see based on their personal preferences in a vast amount of visual information. Based on the analysis results of the attention map in the eye-movement experiment, designers can effectively grasp and utilize the fragmented area of the platform interface to allow users to access information subconsciously, thus increasing the number of attention points of users on the interface. According to the sample analysis and research, it is found that consumers are first attracted by the headline in the interface, but also get richer information from the surrounding text, patterns, and even visible content objects and other attention fragments. The clever use of such inactive information in the design, combined with active attention to textual content, will help users to enrich and improve their cognition of the platform. For the interface design of mobile learning platforms, designers can analyze and summarize what kind of design style and imagery is preferred by certain kinds of user groups, and carry out targeted fragmentation design. Actively use secondary attention fragments, combine common reading styles, cater to users’ attention habits, visualize and visualize the value of the platform content you want to show, and make the design intent accurately and effectively conveyed, to enhance the perception of the platform.

### Comprehensive analysis

The typographic style of the interface is the main content of interface design and the carrier of visual experience design [25]. The study mainly focuses on the visual experience and visual attention of the interface, with more emphasis on its presentation and visual guidance. The subjects were grouped according to different elements in advance, and the analysis and comparison of eye-movement experiments were conducted for their layout design, but no special experiments were conducted for the overall layout. Based on the experimental results, it can be seen that the subjects will choose different layout combinations according to their needs in a targeted manner. Users will construct the corresponding usage in their mind during the process of browsing the platform, which makes the action path of human interaction with the interface and different usage methods will have an impact on the overall experience. To improve the browsing intention of target users, designers should summarize the interface forms preferred by target user groups through detailed user research and make reasonable use of them. While designing the interactive experience, it is also necessary to consider various usage scenarios of the platform, as well as to achieve aesthetic and interesting innovation of the interface through layout innovation on the basis of satisfying functional requirements, setting guide pages, operation step guidance, masking guidance, point reward guidance, etc. to guide users to become familiar with the interface, reduce the time and energy cost of users, and promote online learning behavior to reach. For the interface of mobile learning platform, the designer can appropriately change the layout of the interface, combine the user’s browsing habits and design interactive feedback. By providing the right timing and user-discoverable behavioral feedback, the user can find the correctness of their own behavior and make the user’s emotion high, thus improving the continuity of the user’s motivation. The feedback interface is set up to prompt users to study and to distinguish between completed and unfinished coursework to optimize the visual experience of users. Currently, the layout of similar platforms in the market is becoming more and more homogeneous and has even become a symbol to some extent. Although the interface layout is not the main reference index in the research experiment, it can still be seen from the experimental results that minor changes in interface layout have a greater impact on users’ subjective feelings and browsing intentions, and users are more inclined to choose the layout format that is generally considered to be more suitable for the platform’s positioning. In addition to improving their own interface design capabilities, designers need to understand users’ visual cognitive abilities and design layout features that meet their visual cognitive habits to more effectively communicate their design intentions and thus improve users’ browsing intentions.

### Research limitations

#### Sample study

Since only the top-ranked platforms in terms of user visits were selected as the main research objects, the existing platforms in the market have a wide variety of interfaces. As the existing English mobile learning platforms for college students have a variety of interfaces and many types of active users in the market, a more extensive and detailed study of the products and users is needed to obtain accurate data and more reliable research conclusions, so the number of users and the range of products in this study need to be expanded.

#### Interface design practice

The final improved English mobile learning interface for college students did not realize real online operation, and the background data of users’ periodic learning could not be obtained, so this study could not fully verify the influence of the new version of the interface on the persistence of college students’ visual attention. Through experimental methods such as eye-movement experiments and questionnaires, it was not possible to make a more in-depth metric on the visual attention of the interface. Therefore, in the later study, a more scientific and comprehensive validation system needs to be constructed to evaluate the visual attention of the interface.

#### Experimental data

Small sample size and insufficient depth of research. The subjects selected for this study are college students in a school, so the sample range is relatively small, the data do not reflect comprehensively enough, and the results have certain limitations.

## Conclusion

User interface design is the link between the platform to convey the platform value and display features and is also the key link between the user and the interface. Through eye-movement experiment analysis, the features and design principles for optimizing visual experience design and visual attention are proposed in five aspects of the mobile learning platform interface: color, text, typography, layout, and secondary areas. The empirical study provides an objective basis for the selection and 0improvement of interface design solutions, helps to build a more complete platform interface design evaluation system, and provides more precise design guidance for the improvement of interface design evaluation theory. Due to the limitations, there are a series of shortcomings in this study, such as the lack of dynamic prototype testing, the data obtained from the design prototype experiment alone cannot explain the rationality and usability of the interaction, and the algorithm used for data analysis is also simple. Later, we will further use the current popular algorithms for research, for examples, Gazelle optimization algorithm: a novel nature-inspired metaheuristic optimizer; Dwarf mongoose optimization algorithm: feature selection and enhanced krill herd algorithm for text document clustering; Prairie dog optimization algorithm: unsupervised text feature selection technique based on hybrid particle swarm optimization algorithm with genetic operators for the text clustering; in addition, due to the different browsing habits of the subjects, some of the browsing time is long, which has a small impact on the subsequent experiments, and further research is needed. With the development of the times, mobile learning platform has become an important carrier for people to use the internet to obtain information. The interface design, can raise the user’s visual attention based on a deeper degree of emotional resonance, meet the user’s psychological needs, form a symbol of social communication, create a platform interface with characteristics, and create considerable economic and cultural value.

## References

[1] Xiaoping Huang, Dadi An. (2020). Visual experience design of food packaging based on an eye-movement experiment. Packaging Engineering, 43(06), pp. 204–212.

[2] Jieran Shao, Mei Wang, Shanshan Zhang. (2020). A usability evaluation method for APP interaction process based on implicit measurement. Packaging Engineering, 41(22), pp. 154–160.

[3] Jing Wang, Ding Fang. (2020). Research on the visual experience of food design based on eye-tracking technology. Packaging Engineering, 41(20), pp. 169–173.

[4] KIM T. J., PETITJEAN M. (2021). Atypical Package Design and Product Categorization. Journal of Product Innovation Management, 38(3), pp. 271–287.

[5] Xia M. Y. (2020). Advances in eye-movement and EEG techniques for product imagery evaluation. Packaging Engineering, 41(20), pp. 69–73.

[6] Kang L.J. (2017). Misconceptions and prospects of eye movement experiments in design research-a review based on the current state of domestic research. Decoration, pp.122–123

[7] Rui Sun, Juan Li. (2020). Research on the graphic visual experience of intangible cultural heritage based on eye-tracking. Packaging Engineering, 41(8), pp. 263–268.

[8] Rui Zhou, Binfei Luo. (2016). Analysis of the visual experience of academic journal cover design under the background of "Internet+". Decoration, pp. 85–87.

[9] Xun Li, Linhui Sun, Penye Zhu. (2021). A study on the influence of visual salience of pop-up ads on consumers’ attention. Ergonomics, 27(6), pp.33–40.

[10] Ying Hu. (2021). Research on the influence of icon design elements on visual attention and comfort. Packaging Engineering, 42(6), pp.232–238.

[11] Liu Y.G, Liu Q.Y. (2020).A study on learners’ visual attention to micro learning video text design. Packaging Engineering, 41(18), pp.264–272.

[12] Batubara H. H., Sumantri M. S., & Marini A. (2022). Developing an Android-Based E-Textbook to Improve Learning Media Course Outcomes. International Journal of Interactive Mobile Technologies (iJIM), 16(17), pp. 4–19.

[13] Kabiljagić M., Wachtler J., Ebner M., & Ebner M. (2022). Math Trainer as a Chatbot Via System (Push) Messages for Android. International Journal of Interactive Mobile Technologies (iJIM), 16(17), pp. 75–87.

[14] Ajallouda L., Fagroud F. Z., Zellou A., & Benlahmar E. habib. (2022). A Systematic Literature Review of Keyphrases Extraction Approaches. International Journal of Interactive Mobile Technologies (iJIM), 16(16), pp. 31–58. doi: 10.3991/ijim.v16i16.33081

[15] Ahdab Najib Hijazi, & Ahmad Hanif Ahmad Baharin. (2022). The Effectiveness of Digital Technologies Used for the Visitor’s Experience in Digital Museums. A Systematic Literature Review from the Last Two Decades. International Journal of Interactive Mobile Technologies (iJIM), 16(16), pp. 142–159.

[16] Suryandoko W., Mustaji, Bachri B. S., & Sabri I. (2022). “Ku Pantomime Wellmime” Digital Mobile Learning for Cultural Arts Subjects. International Journal of Interactive Mobile Technologies (iJIM), 16(16), pp. 226–242.

[17] Ariffin S. A., Fathil N. S., Yatim M. H. M., & Samsuri M. Z. (2022). A Review on Cultural Design Elements for Mobile Applications User Interface. International Journal of Interactive Mobile Technologies (iJIM), 16(15), pp. 78–92.

[18] Che Ilias I. S., Ramli S., Wook M., & Hasbullah N. A. (2022). Factors Influencing Users’ Satisfaction Towards Image Use in Social Media: A PLS-SEM Analysis. International Journal of Interactive Mobile Technologies (iJIM), 16(15), pp. 172–186. doi: 10.3991/ijim.v16i15.32555

[19] Mu’azah Md. Aziz, Yusuff Adeola Moshood, & Ainul Maulid Ahmad. (2022). The Effectiveness of Using Application as An Online Basic Computer Learning Medium in Higher Education Institution. International Journal of Interactive Mobile Technologies (iJIM), 16(13), pp. 107–117.

[20] Alsharaiah M. A., Baniata H., L., Al Adwa O., Abu Alghanam O., Abu-Shareha A. A., Alzboon L., et al. (2022). Neural Network Prediction Model to Explore Complex Nonlinear Behavior in Dynamic Biological Network. International Journal of Interactive Mobile Technologies (iJIM), 16(12), pp. 32–51.

[21] NakiÄ‡ J., Burčul A., & MaranguniÄ‡ N. (2019). User-Centred Design in Content Management System Development: The Case of EMasters. International Journal of Interactive Mobile Technologies (iJIM), 13(08), pp. 43–59.

[22] Al Said N., & Al-Said K. M. (2020). Assessment of Acceptance and User Experience of Human-Computer Interaction with a Computer Interface. International Journal of Interactive Mobile Technologies (iJIM), 14(11), pp. 107–125.

[23] Etiyawati N., Dwi Purnomo H., & Mailoa E. (2022). User Experience Design on Visualization of Mobile-Based Land Monitoring System Using a User-Centered Design Approach. International Journal of Interactive Mobile Technologies (iJIM), 16(03), pp. 47–65.

[24] Tolentino J. C. G., Miranda J. P. P., Punzalan R. B., Manalang J. C., Hermogenes L. K. S., & Mallari J. T. (2022). Towards the Development of a Mobile Application in Movement Competency Training Grounded on the User-Centered Design Model: The Case of a State University in the Philippines. International Journal of Interactive Mobile Technologies (iJIM), 16(03), pp. 92–103.

[25] Octaberlina L. R., & Rofiki I. (2021). Using Online Game for Indonesian EFL Learners to Enrich Vocabulary. International Journal of Interactive Mobile Technologies (iJIM), 15(01), pp. 168–183. doi: 10.3991/ijim.v15i01.17513

